# Myelosuppression by sunitinib is flt-3 genotype dependent

**DOI:** 10.1038/sj.bjc.6605813

**Published:** 2010-08-03

**Authors:** N P van Erp, R H J Mathijssen, A A van der Veldt, J B Haanen, A K L Reyners, K Eechoute, E Boven, J A M Wessels, H-J Guchelaar, H Gelderblom

**Affiliations:** 1 Departments of Clinical Pharmacy and Toxicology, Leiden University Medical Center, Leiden, The Netherlands; 2 Department of Medical Oncology, Erasmus University Medical Center, Rotterdam, The Netherlands; 3 Department of Medical Oncology, VU University Medical Center, Amsterdam, The Netherlands; 4 Department of Medical Oncology, The Netherlands Cancer Institute, Antoni van Leeuwenhoek Hospital, Amsterdam, The Netherlands; 5 Department of Medical Oncology, University Medical Center Groningen, Groningen, The Netherlands; 6 Department of Clinical Oncology, Leiden University Medical Center, Leiden, The Netherlands

**Sir,**

Myelosuppression is frequently observed during treatment with the multi tyrosine kinase inhibitors (TKIs) (Motzer *et al*, 2007; Bhojani *et al*, 2008). The frequency and severity of myelosuppression varies among the drug class with sunitinib being a relative myelotoxic compound leading to leukopenia and thrombocytopenia in 56–78% and 41–65% of the patients, respectively (Demetri *et al*, 2006; Motzer *et al*, 2007).

In a previous issue of the British Journal of Cancer, Kumar *et al* (2009) have investigated the potency of three TKIs that target the vascular endothelial growth factor receptor; sunitinib, sorafenib and pazopanib, against a large panel of kinases in *in vitro* and cellular assays. They further evaluated the potential for myelosuppression by measuring the ability of these TKIs to inhibit human bone marrow progenitor growth. Both c-kit and flt-3 seemed to be important kinases for the development of early stem and progenitor cells, as determined by Kumar *et al* (2009). The differences in activity against these kinases provide a plausible explanation for the observed differences in clinical myelosuppression between sunitinib, sorafenib and pazopanib (Hartmann *et al*, 2009; Kumar *et al*, 2009).

As there is considerable variability among patients with regard to sunitinib-induced myelosuppression, additional patient characteristics could very well contribute to the development of bone marrow toxicity. In a recent study, we have investigated the relationship between germ-line variants in genes encoding proteins involved in sunitinib disposition, metabolism and mechanism of action and the development of common toxicities, including haematological toxicities (van Erp *et al*, 2009). We found a strong association between the presence of the flt-3 738C-allele, which is a non-synonymous polymorphism in the flt-3 receptor with currently unknown functionality, and a 2.8-fold reduction in the risk for developing leukopenia after one cycle of sunitinib treatment. This finding not only underlines the role of flt-3 in myelosuppression as suggested by Kumar *et al* (2009) but might also explain the large interpatient variability in sunitinib-related leukopenia response.

In our study, only 29% of the patients developed thrombocytopenia in the first treatment cycle as compared with 46% of the patients who developed leukopenia scored according to the criteria of Common Toxicity Criteria for Adverse Events version 3.0. An explanation for this difference might be the high baseline thrombocyte counts (mean: 319 × 10^9^ l^–1^, range: 92–864 × 10^9^ l^–1^) in these patients. As a consequence, a larger decrease in thrombocytes is necessary to develop thrombocytopenia.

We, therefore, performed a sub-analysis, in 193 patients, in which the thrombocyte count ratios (counts after 4 weeks of treatment/baseline counts) were univariately tested for an association with the flt-3 polymorphism 738T/C. The flt-3 genotype was divided into two groups wild type (TT) (*n*=59) *vs* C-allele carriers (CT/CC) (*n*=134) since a protective effect was observed in C-allele carriers in developing leukopenia in our earlier analysis (van Erp *et al*, 2009). In line with our previous analysis, a protective effect in thrombocyte reduction was indeed observed for the flt-3 738C-allele carriers. After 4 weeks of sunitinib treatment, the mean thrombocyte count ratios for carriers of the flt-3 738 TT genotype *vs* carriers of the flt-3 738 CT/CC genotype were 0.54 *vs* 0.65 (*P*=0.024) (Figure 1).

We conclude that the flt-3 738C allele has a protective effect against sunitinib-induced thrombocytopenia. Combined with our earlier finding with regard to leukopenia it seems that the flt-3 738C>T polymorphism has a role in the variability of sunitinib-induced bone marrow toxicities.

## Figures and Tables

**Figure 1:**
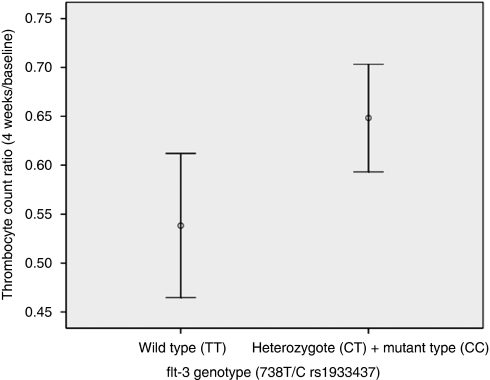
Trombocyte counts decrease after sunitinib start in flt3 738 TT *vs* CT and CC carriers.
